# Supplementary material for: An evaluation of RNA-seq differential analysis methods
Source: PLoS One. 2022 Sep 16;17(9):e0264246. doi: 10.1371/journal.pone.0264246 (PMC9480998; doi:10.1371/journal.pone.0264246)
Supplement: S1 Table — (PDF) [file pone.0264246.s001.pdf]

S1 Table. Estimated FDR of compared RNA-seq differential analysis methods from negative binomial distributed RNA-seq count data.

n	π_1	Estimated FDR with equal library sizes								
		edgeR Exact	edgeR GLM	DESeq	DESeq2	baySeq	EBSeq	SAMSeq	NOISeq	Voom
3	0.01	0.9775	0.9812	0.9895	0.9249	1.0000	0.9314	0.9590	0.9636	1.0000
	0.05	0.9031	0.8961	0.9423	0.6939	0.7172	0.7089	0.7964	0.8203	1.0000
	0.10	0.8021	0.8345	0.7886	0.4981	0.5542	0.5271	0.6112	0.6595	1.0000
	0.25	0.5939	0.6206	0.5734	0.2505	0.3425	0.2749	0.2790	0.4010	1.0000
	0.50	0.2541	0.2763	0.2410	0.1026	0.1224	0.1172	0.1260	0.2145	1.0000
	0.75	0.1222	0.1275	0.1234	0.0412	0.0514	0.0482	0.0925	0.0994	0.3750
	0.90	0.0303	0.0355	0.0094	0.0104	0.0066	0.0124	0.0406	0.0244	0.0000
6	0.01	1.0000	1.0000	1.0000	0.6113	0.2030	0.7116	0.5336	–	0.5000
	0.05	1.0000	1.0000	1.0000	0.2946	0.0800	0.3093	0.2558	0.0000	0.0714
	0.10	0.6154	0.5294	–	0.1878	0.0633	0.2212	0.0873	0.2000	0.0727
	0.25	0.3023	0.2464	0.4000	0.1038	0.0378	0.1073	0.0703	0.0357	0.0727
	0.50	0.0971	0.0916	0.2143	0.0464	0.0128	0.0442	0.0553	0.0119	0.0381
	0.75	0.0412	0.0388	0.0000	0.0235	0.0128	0.0182	0.0923	0.0109	0.0304
	0.90	0.0037	0.0080	0.0000	0.0099	0.0028	0.0063	0.0734	0.0000	0.0198
12	0.01	0.0000	0.2013	–	0.1209	0.0000	0.2804	0.0000	0.0000	0.0595
	0.05	0.1512	0.1461	1.0000	0.1698	0.0625	0.1038	0.0849	0.1000	0.0476
	0.10	0.0472	0.0526	0.0000	0.0725	0.0000	0.0426	0.0294	0.0400	0.0229
	0.25	0.0417	0.0431	–	0.0535	0.0180	0.0340	0.0566	0.0240	0.0989
	0.50	0.0287	0.0351	0.0200	0.0341	0.0075	0.0154	0.0366	0.0206	0.0338
	0.75	0.0175	0.0198	0.0110	0.0199	0.0055	0.0095	0.1004	0.0211	0.0802
	0.90	0.0064	0.0082	0.0041	0.0143	0.0021	0.0046	0.0660	0.0081	0.0398
n	π_1	Estimated FDR with unequal library sizes								
		edgeR Exact	edgeR GLM	DESeq	DESeq2	baySeq	EBSeq	SAMSeq	NOISeq	Voom
3	0.01	0.9969	0.9853	0.9919	0.9207	0.9467	0.9301	0.9640	0.9719	–
	0.05	0.9271	0.8965	0.8337	0.6791	0.7848	0.7012	0.8067	0.8155	–
	0.10	0.8343	0.8287	0.7644	0.4537	0.5902	0.5254	0.6225	0.6416	1.0000
	0.25	0.6526	0.5938	0.5292	0.2285	0.3570	0.2589	0.2520	0.3992	0.0000
	0.50	0.3115	0.3173	0.2020	0.1003	0.1405	0.1165	0.1567	0.2152	1.0000
	0.75	0.1488	0.1446	0.2417	0.0422	0.0473	0.0501	0.1211	0.1051	–
	0.90	0.0322	0.0301	0.0433	0.0103	0.0090	0.0132	0.0419	0.0262	0.0000
6	0.01	1.0000	1.0000	1.0000	0.5807	0.4950	0.6900	0.5029	–	0.4000
	0.05	0.5000	0.6667	–	0.2479	0.0385	0.3304	0.2571	0.3333	0.0370
	0.10	0.6000	0.6000	–	0.1577	0.0435	0.1643	0.0735	0.1429	0.0400
	0.25	0.4286	0.4211	0.0000	0.0963	0.0043	0.0741	0.0436	0.0000	0.0421
	0.50	0.0781	0.1304	0.5000	0.0487	0.0236	0.0405	0.0377	0.0549	0.0322
	0.75	0.0798	0.0756	0.0909	0.0291	0.0100	0.0213	0.1119	0.0220	0.0379
	0.90	0.0057	0.0061	0.0000	0.0073	0.0009	0.0040	0.0624	0.0000	0.0133
12	0.01	0.3333	0.3333	1.0000	0.1161	0.0008	0.3344	0.0485	–	0.0619
	0.05	0.1294	0.1379	1.0000	0.1429	0.0361	0.1351	0.0614	0.1111	0.0446
	0.10	0.0772	0.0789	–	0.1117	0.0146	0.0970	0.0498	0.0000	0.0519
	0.25	0.0438	0.0500	0.0000	0.0581	0.0165	0.0342	0.0560	0.0233	0.0747
	0.50	0.0291	0.0311	0.0090	0.0352	0.0065	0.0153	0.0534	0.0305	0.0260
	0.75	0.0141	0.0172	0.0196	0.0222	0.0049	0.0039	0.1056	0.0280	0.0759
	0.90	0.0040	0.0054	0.0038	0.0120	0.0016	0.0024	0.0778	0.0068	0.0262
