# Supplementary material for: An evaluation of RNA-seq differential analysis methods
Source: PLoS One. 2022 Sep 16;17(9):e0264246. doi: 10.1371/journal.pone.0264246 (PMC9480998; doi:10.1371/journal.pone.0264246)
Supplement: S3 Table — (PDF) [file pone.0264246.s003.pdf]

S3 Table. Estimated FDR and power of compared RNA-seq differential analysis methods from log-normal distributed RNA-seq count data.

n	π_1	Estimated FDR with unequal library sizes								
		edgeR Exact	edgeR GLM	DESeq	DESeq2	baySeq	EBSeq	SAMSeq	NOISeq	Voom
3	0.05	0.0860	0.1542	0.0083	0.0130	0.0341	0.0203	0.0844	0.3513	0.9569
	0.10	0.0636	0.1004	0.0111	0.0139	0.0180	0.0170	0.0551	0.0724	0.9041
	0.15	0.0483	0.0588	0.0078	0.0121	0.0216	0.0093	0.0648	0.0450	0.8567
	0.20	0.0316	0.0415	0.0121	0.0117	0.0203	0.0101	0.0577	0.0278	0.8131
6	0.05	0.0040	0.2257	0.0000	0.0000	0.0021	0.0000	0.1675	0.0655	0.9528
	0.10	0.0364	0.1406	0.0161	0.0000	0.0000	0.0000	0.1111	0.0000	0.9001
	0.15	0.0220	0.0962	0.0105	0.0105	0.0106	0.0000	0.0885	0.0187	0.8449
	0.20	0.0160	0.0752	0.0081	0.0161	0.0161	0.0000	0.0699	0.0219	0.7974
12	0.05	0.0402	0.1087	0.0000	0.0385	0.0385	0.0418	0.0965	0.0306	0.9514
	0.10	0.0159	0.0462	0.0000	0.0145	0.0154	0.0172	0.0769	0.0137	0.8979
	0.15	0.0100	0.0291	0.0000	0.0099	0.0096	0.0106	0.0427	0.0089	0.8418
	0.20	0.0078	0.0229	0.0000	0.0074	0.0075	0.0085	0.0516	0.0071	0.7940
n	π_1	Estimated power with unequal library sizes								
		edgeR Exact	edgeR GLM	DESeq	DESeq2	baySeq	EBSeq	SAMSeq	NOISeq	Voom
3	0.05	0.7249	0.7149	0.7428	0.7201	0.7314	0.6076	0.7253	0.8946	0.9838
	0.10	0.6674	0.6710	0.8226	0.6890	0.6631	0.5541	0.8810	0.9524	0.9719
	0.15	0.7932	0.7970	0.9116	0.8273	0.8062	0.6635	0.9282	0.9690	0.9699
	0.20	0.8143	0.8466	0.9115	0.8429	0.8338	0.6920	0.9160	0.9505	0.9749
6	0.05	0.6216	0.6216	0.6216	0.6216	0.6216	0.5946	0.6500	0.7270	1.0000
	0.10	0.6625	0.6875	0.7625	0.7250	0.6750	0.5875	0.8000	0.8375	0.9750
	0.15	0.7177	0.7581	0.7581	0.7581	0.7500	0.6613	0.8306	0.8468	0.9758
	0.20	0.7688	0.7688	0.7625	0.7625	0.7625	0.7063	0.8313	0.8375	0.9875
12	0.05	0.6402	0.6402	0.5915	0.6402	0.6402	0.5878	0.7196	0.7683	1.0000
	0.10	0.7470	0.7470	0.8193	0.8193	0.7711	0.6867	0.8675	0.8675	0.9880
	0.15	0.7674	0.7752	0.8140	0.7752	0.7984	0.7209	0.8682	0.8605	0.9845
	0.20	0.7560	0.7619	0.8095	0.8036	0.7917	0.6964	0.8750	0.8333	0.9881
