# Supplementary material for: An evaluation of RNA-seq differential analysis methods
Source: PLoS One. 2022 Sep 16;17(9):e0264246. doi: 10.1371/journal.pone.0264246 (PMC9480998; doi:10.1371/journal.pone.0264246)
Supplement: Attachment — Submitted filename: Responses to Reviewers.docx [file pone.0264246.s004.docx]

**Overview of responses to reviewers**

**Manuscript ID: PONE-D-22-03667**

**An evaluation of RNA-seq differential analysis methods**

**PLOS ONE**

**We appreciate the many suggestions and comments from the reviewers. We have revised our manuscript to incorporate reviewers’ comments and suggestions. The details are listed below.**

*5. Review Comments to the Author

Please use the space provided to explain your answers to the questions above. You may also include additional comments for the author, including concerns about dual publication, research ethics, or publication ethics. (Please upload your review as an attachment if it exceeds 20,000 characters)*

*Reviewer #1: Li et al have produced a robust and thorough review of differential expression analysis tools. The article is very well written, included all technical descriptions of the tools and methods they employ and gives a well reasoned discussion and conclusion for each.

I have a few very minor things which I would like to see addressed:

On line 42 you describe DeSeq2 as recent, I would disagree as it was published in 2014. I think this needs rewording.*

**Response: Thanks for the suggestions. We agree with the reviewer and have reworded the sentence in our revised manuscript.**

**“DESeq2 is a successor to the DESeq method with flexibility to accommodate more complexed study design of sequencing experiments”.**

*I think in the introduction more emphasis could be made on previous published comparisons being 8-10 years old and it being time for an update.*

**Response: Great suggestions. We have emphasized that the previous published comparisons are 8-10 in our revised manuscript.**

**“As more statistical methods for gene differential analyses have been developed in recent years, there is a need for an update on the differential analysis methods comparisons”.**

*I think a layman's description of Power and Stability would be of benefit to newer bioinformaticians and would widen the scope of the readership.*

**Response: Great suggestions. We have added the description of Power and Stability using layman language in our revised manuscript.**

**“Power is defined as the expected proportion of identified differentially expressed genes among all the truly differentially expressed genes, given at least one gene is truly differentially expressed in the data. Stability is defined as the standard deviation of total rejections.”**

*Figure 3 axis titles are a bit squashed compared to others, would be nice to see them consistent (however this may be an artefact of the submission).*

**Response: Thanks for the suggestions. We have revised Figure 3 axis titles to make them consistent with other Figure axis titles.** *Figure 4 legend needs to include A and B descriptions to match the figure.*

**Response: Thanks for the suggestions. A and B descriptions are added.** *Finally why has Sleuth been committed? Rationale needs adding to discussion on choice of tools. As "popular" depends on the audience.*

**Response: Thanks for the suggestions. We have added this into our limitations in the Discussion section as the methods we chose are “popular” methods in our opinion, which might not be considered as “popular” methods by others.**

**“Another limitation is that we only compared the gene differential analysis methods that fall in our interest. Our current methods comparison might miss some new methods (such as the Sleuth method) that are getting popular in gene differential analyses. Future updated comparisons of RNA-seq differential analysis methods are needed to incorporate newly developed methods that are getting popular in recent years”.**

*Reviewer #2: The authors have conducted an interesting study comparing popular tools for gene differential expression analysis. Using a combination of simulations and RNA-seq data from mice they compare the power, FDR and stability of results for each tool.

The results are clearly presented and address relevant questions for RNA-seq analysts.

The discussion would benefit from some comments on why the number of DE genes vary so much among the negative-binomial models in the mouse data. Why does EdgeR rank amongst the lowest for power in the simulations at n=6 and n=3 samples, but ranks highly for number of significant genes identified in the real data?*

**Response: Thanks for the suggestions. We have added comments on why the number of DE genes vary so much among the negative-binomial models in the mouse data as well as why EdgeR rank amongst the lowest for power in the simulations at n = 6 and n = 3 samples but ranks highly for number of significant genes identified in the real data.**

**“We noticed that the number of differentially expressed genes identified from the real data have large variations among the negative-binomial based models. This large variation might be due to the different ways of estimating the standard deviations in the models, which lead to the power differences in different models. We also observed that edgeR ranks amongst the lowest for power in the simulations at n = 6 and n = 3 sample per group, while edgeR ranks highly for number of significant genes identified in the real data. This difference might be due to the distribution differences between the simulated data and real data. Our simulated data assumes independence between genes, which might not be the situation in the real data.”** *The discussion also needs to mention the limitations of the simulations used. In particular, random sampling from negative-binomial and log-normal distributions assumes independence between samples, which is unrealistic in the case of gene expression. This approach does not take the structural correlation among genes into account.*

**Response: Thanks for the comments. We have added the limitation of our simulation study in the discussion section in our revised manuscript.**

**“A limitation of our simulation study is the lack of consideration of structural correlation among genes as our random sampling from negative-binomial and log-normal distributions assumes independence between samples, which is not the real situation in gene expression. Future simulations considering the gene correlations are needed to further evaluate the performance of RNA-seq differential analyses methods. ”** *It would also be appreciated if the code and simulation data could be made available on a public repository, such as Zenodo or Github. Having it available upon request by email introduces an unnecessary hurdle, especially in light of the recent paper by Gabelica et al.* [*https://doi.org/10.1016/j.jclinepi.2022.05.019*](https://urldefense.proofpoint.com/v2/url?u=https-3A__doi.org_10.1016_j.jclinepi.2022.05.019&d=DwQGaQ&c=4sF48jRmVAe_CH-k9mXYXEGfSnM3bY53YSKuLUQRxhA&r=azjhQW59umxfk67jsIXeg7AS2ugqKLkykGYJFRwaLto&m=jU3utht8y489P4H19xzUe6yfP8XSw3VplvHD-j-pBVw&s=azj-1wMF_jcVK0EY6Z_oJM4xdDCi5GPMjw4BHnAnIXw&e=)

**Response: Thanks for the suggestions. We have deposited the simulation data and code into Github. It can be accessed at** [**https://github.com/DongmeiLi2017/RNA-seq-Analysis-Methods-Comparison**](https://github.com/DongmeiLi2017/RNA-seq-Analysis-Methods-Comparison)**.**

*Some minor points to address:

Line 240 — "have very little power and reject very few genes" doesn't make sense here, please clarify. Does this refer to rejecting hypotheses?*

**Response: Thanks for the comments. It refers to rejecting hypotheses. We have changed it to “have very little power and reject very few hypotheses.”** *Line 341 — "the sample size is 12 in each group" doesn't fit with Figure 4a containing n=21 total. For consistency with Figures 1-3, it would be clearer to stick to the "n=samples per group" format rather than "n=total samples".*

**Response: Thanks for the comments. We have changed for format to “n = samples per group” in our revised manuscript.**
